# (2-Hy­droxy-4-meth­oxy­phen­yl)(2-hy­droxy­phen­yl)methanone

**DOI:** 10.1107/S1600536811030042

**Published:** 2011-07-30

**Authors:** Richard Betz, Thomas Gerber, Eric Hosten, Henk Schalekamp

**Affiliations:** a Nelson Mandela Metropolitan University, Summerstrand Campus, Department of Chemistry, University Way, Summerstrand, PO Box 77000, Port Elizabeth 6031, South Africa

## Abstract

The title compound, C_14_H_12_O_4_, is an asymmetric substitution product of benzophenone. Both hy­droxy groups are orientated towards the O atom of the keto group. Intra­molecular as well as inter­molecular O—H⋯O hydrogen bonds can be observed in the crystal structure, with the latter connecting the mol­ecules into chains along the crystallographic *b* axis. C—H⋯O contacts [C⋯O = 3.3297 (18) Å] are also apparent. The closest centroid–centroid distance between two aromatic systems is 4.9186 (9) Å.

## Related literature

For the crystal structure of benzophenone, see: Lobanova (1968 ▶); Kutzke *et al.* (2000 ▶); Fleischer *et al.* (1968 ▶); Bernstein *et al.* (2002 ▶); Moncol & Coppens (2004 ▶). For the crystal structure of bis­(2-hy­droxy­phen­yl)methanone, see: Betz *et al.* (2011 ▶). For details on graph-set analysis of hydrogen bonds, see: Etter *et al.* (1990 ▶); Bernstein *et al.* (1995 ▶). For a comparison of the thermodynamic stability of coordination compounds containing chelate ligands as opposed to monodentate ligands, see: Gade (1998 ▶).
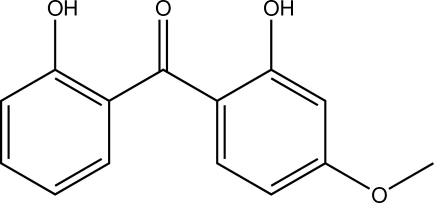


## Experimental

### 

#### Crystal data

C_14_H_12_O_4_
*M* *_r_* = 244.24
Orthorhombic, 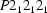

*a* = 4.8582 (2) Å
*b* = 14.0236 (5) Å
*c* = 16.8636 (5) Å
*V* = 1148.91 (7) Å^3^
*Z* = 4
Mo *K*α radiation
μ = 0.10 mm^−1^
*T* = 200 K
0.48 × 0.14 × 0.05 mm

#### Data collection

Bruker APEXII CCD diffractometer
6314 measured reflections
1683 independent reflections
1484 reflections with *I* > 2σ(*I*)
*R*_int_ = 0.021

#### Refinement

*R*[*F* ^2^ > 2σ(*F* ^2^)] = 0.030
*wR*(*F* ^2^) = 0.083
*S* = 1.07
1683 reflections
166 parameters
H-atom parameters constrained
Δρ_max_ = 0.18 e Å^−3^
Δρ_min_ = −0.17 e Å^−3^

### 

Data collection: *APEX2* (Bruker, 2010 ▶); cell refinement: *SAINT* (Bruker, 2010 ▶); data reduction: *SAINT*; program(s) used to solve structure: *SHELXS97* (Sheldrick, 2008 ▶); program(s) used to refine structure: *SHELXL97* (Sheldrick, 2008 ▶); molecular graphics: *ORTEP-3* (Farrugia, 1997 ▶) and *Mercury* (Macrae *et al.*, 2008 ▶); software used to prepare material for publication: *SHELXL97* and *PLATON* (Spek, 2009 ▶).

## Supplementary Material

Crystal structure: contains datablock(s) I, global. DOI: 10.1107/S1600536811030042/lw2071sup1.cif

Supplementary material file. DOI: 10.1107/S1600536811030042/lw2071Isup2.cdx

Structure factors: contains datablock(s) I. DOI: 10.1107/S1600536811030042/lw2071Isup3.hkl

Supplementary material file. DOI: 10.1107/S1600536811030042/lw2071Isup4.cml

Additional supplementary materials:  crystallographic information; 3D view; checkCIF report

## Figures and Tables

**Table 1:** Hydrogen-bond geometry (Å, °)

*D*—H⋯*A*	*D*—H	H⋯*A*	*D*⋯*A*	*D*—H⋯*A*
O2—H2⋯O1	0.84	1.88	2.6058 (17)	144
O3—H3⋯O1	0.84	1.91	2.6267 (17)	142
O3—H3⋯O4^i^	0.84	2.50	2.9306 (15)	113
C15—H15⋯O1^ii^	0.95	2.57	3.3297 (18)	137

Symmetry codes: (i) 
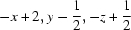
; (ii) 
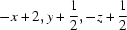
.

**Fig. 1.**
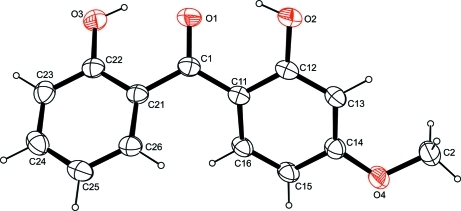
The molecular structure of the title compound, with atom labels and anisotropic displacement ellipsoids (drawn at 50% probability level).

**Fig. 2.**
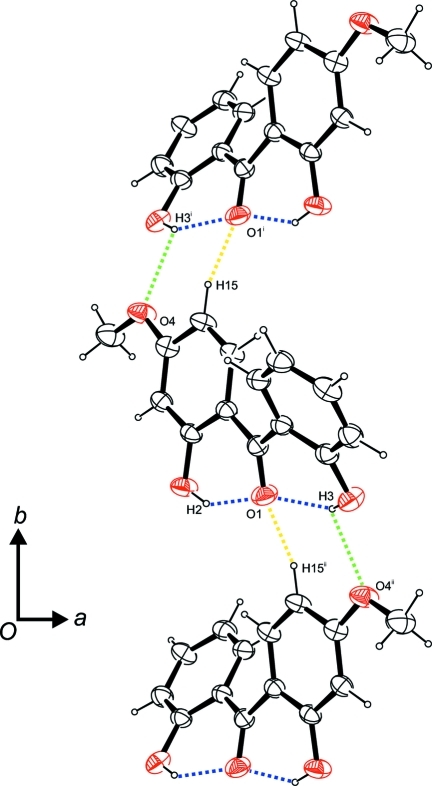
Intermolecular contacts, viewed along [0 0 - 1]. Blue dashed lines indicate intramolecular hydrogen bonds, green dashed lines intermolecular hydrogen bonds and yellow dashed lines C–H···O contacts. Symmetry operators: i -x + 2, y + 1/2, -z + 1/2; ii -x + 2, y - 1/2, -z + 1/2.

**Fig. 3.**
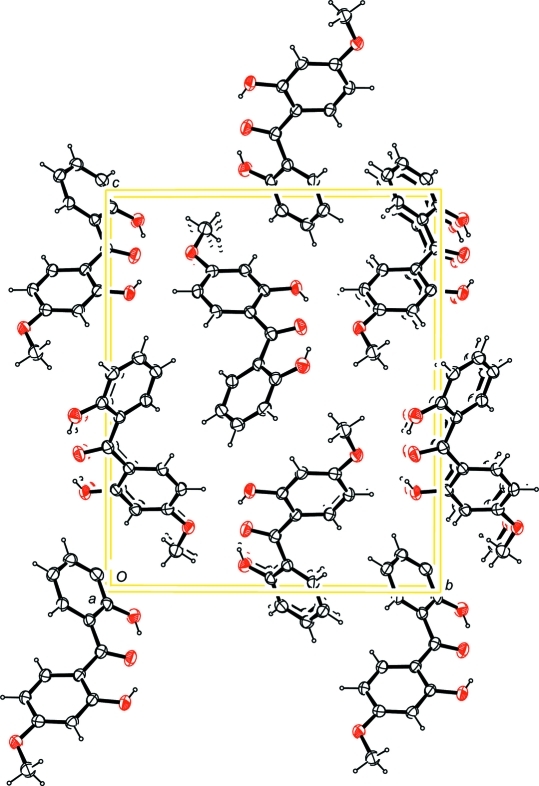
Molecular packing of the title compound, viewed along [-1 0 0] (anisotropic displacement ellipsoids drawn at 50% probability level).

## supplementary crystallographic information

### Comment

Chelate ligands have found widespread use in coordination chemistry due to the enhanced thermodynamic stability of resultant coordination compounds in relation to coordination compounds exclusively applying comparable monodentate ligands (Gade, 1998). Combining two identical donor atoms in different states of hybridization seemed to be useful to us to accomodate a large variety of metal centers of variable Lewis acidity. To enable comparative studies in terms of bond lengths and angles in envisioned coordination compounds, we determined the molecular and crystal structure of the title compound. The crystal structure of benzophenone is apparent in the literature (Lobanova, 1968; Kutzke *et al.*, 2000; Fleischer *et al.*, 1968; Bernstein *et al.*, 2002; Moncol & Coppens, 2004) as is the crystal structure of bis(2-hydroxyphenyl)methanone (Betz *et al.*, 2011).

The title compound is an asymmetric substitution product of benzophenone. Both aromatic moieties adopt a conformation in which its hydroxyl group is orientated towards the central oxygen atom. The least-squares planes defined by the respective carbon atoms of both aromatic rings intersect at an angle of 42.11 (6) °. Intracyclic C–C–C angles hardly deviate from the ideal value of 120 °. The methoxy group is nearly in plane with its resident aromatic system, the respective C–O–C–C torsional angle is found at 4.9 (2) ° (Fig. 1).

In the crystal structure, intra- as well as intermolecular hydrogen bonds are observed. While the intramolecular hydrogen bonds are apparent between the hydroxyl groups as donors and the double-bonded oxygen atom as acceptor, the intermolecular hydrogen bond stems from the hydroxyl group on the otherwise unsubstituted phenyl ring and has the etheric oxygen atom as acceptor (Fig. 2). The latter hydrogen bond thus shows bifurcation. In addition, a C–H···O contact whose range falls by more than 0.1 Å below the sum of van-der-Waals radii is present in the crystal structure. The latter one is supported by one of the CH groups in *ortho*-position to the methoxy substituent and has the keto group's oxygen atom as acceptor. In terms of graph-set analysis (Etter *et al.*, 1990; Bernstein *et al.*, 1995), the descriptor for the hydrogen bonding system based on the hydroxyl groups on the unitary level is *S*(6)*S*(6)*C*(10) while the C–H···O contacts necessitate a *C*(6) descriptor on the same level. In total, the molecules are connected to undulated chains along the crystallographic *b* axis. The shortest intercentroid distance between two aromatic systems was measured to be at 4.9186 (9) Å and is apparent between the two different aromatic moieties.

The molecular packing of the title compound in the crystal structure is shown in Figure 3.

### Experimental

The compound was obtained commercially (Aldrich). Crystals suitable for the X-ray diffraction study were taken directly from the provided product.

### Refinement

Carbon-bound H atoms were placed in calculated positions (C—H 0.95 Å for aromatic carbon atoms) and were included in the refinement in the riding model approximation, with *U*(H) set to 1.2*U*_eq_(C). The H atoms of the methyl group were allowed to rotate with a fixed angle around their respective C—O bond to best fit the experimental electron density (HFIX 137 in the *SHELX* program suite (Sheldrick, 2008)), with *U*(H) set to 1.5*U*_eq_(C) and C—H set to 0.98 Å. The H atoms of the hydroxyl groups were allowed to rotate with a fixed angle around their respective C—O bond to best fit the experimental electron density (HFIX 147 in the *SHELX* program suite (Sheldrick, 2008)), with *U*(H) set to 1.5*U*_eq_(O) and O—H set to 0.84 Å.

### Crystal data

**Table:**

C_14_H_12_O_4_	*F*(000) = 512
*M**_r_* = 244.24	*D*_x_ = 1.412 Mg m^−^^3^
Orthorhombic, *P*2_1_2_1_2_1_	Mo *K*α radiation, λ = 0.71073 Å
Hall symbol: P 2ac 2ab	Cell parameters from 4306 reflections
*a* = 4.8582 (2) Å	θ = 2.8–28.2°
*b* = 14.0236 (5) Å	µ = 0.10 mm^−^^1^
*c* = 16.8636 (5) Å	*T* = 200 K
*V* = 1148.91 (7) Å^3^	Platelet, yellow
*Z* = 4	0.48 × 0.14 × 0.05 mm

### Data collection

**Table:**

Bruker APEXII CCD diffractometer	1484 reflections with *I* > 2σ(*I*)
Radiation source: fine-focus sealed tube	*R*_int_ = 0.021
graphite	θ_max_ = 28.3°, θ_min_ = 1.9°
φ and ω scans	*h* = −4→6
6314 measured reflections	*k* = −18→17
1683 independent reflections	*l* = −21→22

### Refinement

**Table:**

Refinement on *F*^2^	Primary atom site location: structure-invariant direct methods
Least-squares matrix: full	Secondary atom site location: difference Fourier map
*R*[*F*^2^ > 2σ(*F*^2^)] = 0.030	Hydrogen site location: inferred from neighbouring sites
*wR*(*F*^2^) = 0.083	H-atom parameters constrained
*S* = 1.07	*w* = 1/[σ^2^(*F*_o_^2^) + (0.0509*P*)^2^ + 0.0857*P*] where *P* = (*F*_o_^2^ + 2*F*_c_^2^)/3
1683 reflections	(Δ/σ)_max_ < 0.001
166 parameters	Δρ_max_ = 0.18 e Å^−^^3^
0 restraints	Δρ_min_ = −0.17 e Å^−^^3^

### Special details

**Table:**

Refinement. Due to the absence of a strong anomalous scatterer, the Flack parameter is meaningless. Thus, Friedel opposites (1966 pairs) have been merged and the item was removed from the CIF.

### Fractional atomic coordinates and isotropic or equivalent isotropic displacement parameters (Å^2^)

**Table:**

	*x*	*y*	*z*	*U*_iso_*/*U*_eq_	
O1	1.0688 (3)	−0.07918 (7)	0.34393 (6)	0.0382 (3)	
O2	0.6572 (3)	−0.06852 (7)	0.24525 (6)	0.0374 (3)	
H2	0.7719	−0.0968	0.2741	0.056*	
O3	1.4961 (3)	−0.08333 (8)	0.43931 (7)	0.0390 (3)	
H3	1.4061	−0.1013	0.3995	0.058*	
O4	0.4289 (3)	0.24323 (8)	0.15520 (6)	0.0405 (3)	
C1	1.0352 (3)	0.00668 (10)	0.36067 (8)	0.0288 (3)	
C2	0.2546 (5)	0.20454 (13)	0.09489 (11)	0.0447 (5)	
H2A	0.3649	0.1660	0.0583	0.067*	
H2B	0.1664	0.2567	0.0657	0.067*	
H2C	0.1130	0.1644	0.1194	0.067*	
C11	0.8770 (3)	0.06797 (10)	0.30686 (8)	0.0273 (3)	
C12	0.6941 (3)	0.02686 (10)	0.25159 (8)	0.0279 (3)	
C13	0.5372 (4)	0.08324 (11)	0.20056 (8)	0.0296 (3)	
H13	0.4108	0.0546	0.1648	0.036*	
C14	0.5676 (4)	0.18145 (10)	0.20262 (8)	0.0309 (3)	
C15	0.7562 (4)	0.22409 (10)	0.25446 (8)	0.0335 (4)	
H15	0.7797	0.2913	0.2545	0.040*	
C16	0.9063 (4)	0.16846 (10)	0.30496 (8)	0.0309 (3)	
H16	1.0341	0.1980	0.3398	0.037*	
C21	1.1541 (4)	0.04328 (10)	0.43571 (8)	0.0275 (3)	
C22	1.3752 (4)	−0.00534 (10)	0.47108 (9)	0.0297 (3)	
C23	1.4840 (4)	0.02689 (11)	0.54312 (9)	0.0344 (4)	
H23	1.6380	−0.0046	0.5659	0.041*	
C24	1.3687 (4)	0.10414 (12)	0.58103 (9)	0.0373 (4)	
H24	1.4441	0.1256	0.6298	0.045*	
C25	1.1439 (4)	0.15086 (11)	0.54881 (9)	0.0355 (4)	
H25	1.0621	0.2030	0.5760	0.043*	
C26	1.0397 (4)	0.12097 (10)	0.47670 (8)	0.0310 (3)	
H26	0.8872	0.1537	0.4543	0.037*	

### Atomic displacement parameters (Å^2^)

**Table:**

	*U*^11^	*U*^22^	*U*^33^	*U*^12^	*U*^13^	*U*^23^
O1	0.0427 (7)	0.0264 (5)	0.0456 (6)	0.0066 (6)	−0.0057 (6)	−0.0090 (4)
O2	0.0490 (7)	0.0230 (5)	0.0403 (5)	−0.0041 (5)	−0.0070 (6)	−0.0036 (4)
O3	0.0417 (7)	0.0346 (6)	0.0406 (6)	0.0111 (6)	−0.0018 (6)	−0.0034 (5)
O4	0.0549 (8)	0.0284 (5)	0.0380 (5)	−0.0066 (6)	−0.0164 (7)	0.0015 (4)
C1	0.0276 (8)	0.0267 (6)	0.0322 (6)	−0.0003 (6)	0.0032 (7)	−0.0035 (5)
C2	0.0554 (12)	0.0360 (8)	0.0428 (8)	−0.0058 (9)	−0.0199 (10)	−0.0001 (7)
C11	0.0292 (8)	0.0252 (7)	0.0275 (6)	−0.0020 (7)	0.0037 (7)	−0.0036 (5)
C12	0.0318 (8)	0.0246 (7)	0.0272 (6)	−0.0045 (6)	0.0057 (7)	−0.0044 (5)
C13	0.0323 (8)	0.0286 (7)	0.0279 (6)	−0.0055 (7)	−0.0010 (7)	−0.0035 (5)
C14	0.0367 (9)	0.0300 (7)	0.0260 (6)	−0.0027 (7)	−0.0003 (8)	0.0006 (5)
C15	0.0449 (9)	0.0231 (6)	0.0324 (7)	−0.0090 (7)	−0.0045 (9)	0.0002 (5)
C16	0.0370 (9)	0.0260 (7)	0.0298 (6)	−0.0080 (7)	−0.0016 (8)	−0.0024 (5)
C21	0.0302 (8)	0.0234 (6)	0.0289 (6)	−0.0021 (6)	0.0037 (7)	0.0008 (5)
C22	0.0324 (8)	0.0254 (7)	0.0313 (6)	−0.0024 (7)	0.0057 (7)	0.0026 (5)
C23	0.0356 (9)	0.0337 (8)	0.0338 (7)	−0.0041 (7)	−0.0028 (8)	0.0057 (6)
C24	0.0470 (10)	0.0348 (8)	0.0300 (7)	−0.0118 (8)	−0.0009 (8)	−0.0011 (6)
C25	0.0436 (10)	0.0285 (7)	0.0343 (7)	−0.0038 (7)	0.0063 (8)	−0.0059 (6)
C26	0.0343 (9)	0.0258 (7)	0.0329 (7)	−0.0011 (7)	0.0022 (7)	−0.0007 (5)

### Geometric parameters (Å, °)

**Table:**

O1—C1	1.2475 (18)	C13—H13	0.9500
O2—C12	1.3537 (16)	C14—C15	1.401 (2)
O2—H2	0.8400	C15—C16	1.366 (2)
O3—C22	1.3522 (19)	C15—H15	0.9500
O3—H3	0.8400	C16—H16	0.9500
O4—C14	1.3578 (19)	C21—C26	1.405 (2)
O4—C2	1.430 (2)	C21—C22	1.405 (2)
C1—C11	1.467 (2)	C22—C23	1.400 (2)
C1—C21	1.483 (2)	C23—C24	1.377 (2)
C2—H2A	0.9800	C23—H23	0.9500
C2—H2B	0.9800	C24—C25	1.384 (3)
C2—H2C	0.9800	C24—H24	0.9500
C11—C12	1.411 (2)	C25—C26	1.382 (2)
C11—C16	1.417 (2)	C25—H25	0.9500
C12—C13	1.395 (2)	C26—H26	0.9500
C13—C14	1.386 (2)		
			
C12—O2—H2	109.5	C16—C15—C14	119.64 (13)
C22—O3—H3	109.5	C16—C15—H15	120.2
C14—O4—C2	118.06 (12)	C14—C15—H15	120.2
O1—C1—C11	119.60 (13)	C15—C16—C11	121.91 (15)
O1—C1—C21	118.44 (14)	C15—C16—H16	119.0
C11—C1—C21	121.95 (12)	C11—C16—H16	119.0
O4—C2—H2A	109.5	C26—C21—C22	118.02 (13)
O4—C2—H2B	109.5	C26—C21—C1	122.29 (15)
H2A—C2—H2B	109.5	C22—C21—C1	119.48 (13)
O4—C2—H2C	109.5	O3—C22—C23	116.15 (15)
H2A—C2—H2C	109.5	O3—C22—C21	123.81 (13)
H2B—C2—H2C	109.5	C23—C22—C21	120.04 (14)
C12—C11—C16	117.06 (14)	C24—C23—C22	120.20 (17)
C12—C11—C1	119.94 (12)	C24—C23—H23	119.9
C16—C11—C1	122.95 (14)	C22—C23—H23	119.9
O2—C12—C13	116.03 (13)	C23—C24—C25	120.74 (15)
O2—C12—C11	122.64 (13)	C23—C24—H24	119.6
C13—C12—C11	121.33 (13)	C25—C24—H24	119.6
C14—C13—C12	119.32 (14)	C26—C25—C24	119.38 (15)
C14—C13—H13	120.3	C26—C25—H25	120.3
C12—C13—H13	120.3	C24—C25—H25	120.3
O4—C14—C13	124.52 (14)	C25—C26—C21	121.54 (16)
O4—C14—C15	114.82 (13)	C25—C26—H26	119.2
C13—C14—C15	120.64 (15)	C21—C26—H26	119.2
			
O1—C1—C11—C12	20.0 (2)	C12—C11—C16—C15	2.8 (2)
C21—C1—C11—C12	−159.02 (14)	C1—C11—C16—C15	−179.82 (15)
O1—C1—C11—C16	−157.31 (16)	O1—C1—C21—C26	−152.15 (16)
C21—C1—C11—C16	23.7 (2)	C11—C1—C21—C26	26.9 (2)
C16—C11—C12—O2	177.13 (15)	O1—C1—C21—C22	22.6 (2)
C1—C11—C12—O2	−0.3 (2)	C11—C1—C21—C22	−158.41 (14)
C16—C11—C12—C13	−3.7 (2)	C26—C21—C22—O3	177.31 (15)
C1—C11—C12—C13	178.86 (14)	C1—C21—C22—O3	2.4 (2)
O2—C12—C13—C14	−178.84 (15)	C26—C21—C22—C23	−3.2 (2)
C11—C12—C13—C14	1.9 (2)	C1—C21—C22—C23	−178.13 (14)
C2—O4—C14—C13	−4.9 (2)	O3—C22—C23—C24	−178.06 (15)
C2—O4—C14—C15	173.44 (16)	C21—C22—C23—C24	2.4 (2)
C12—C13—C14—O4	179.11 (14)	C22—C23—C24—C25	0.1 (3)
C12—C13—C14—C15	0.9 (2)	C23—C24—C25—C26	−1.8 (3)
O4—C14—C15—C16	179.84 (15)	C24—C25—C26—C21	0.9 (2)
C13—C14—C15—C16	−1.8 (3)	C22—C21—C26—C25	1.6 (2)
C14—C15—C16—C11	−0.1 (3)	C1—C21—C26—C25	176.35 (14)

### Hydrogen-bond geometry (Å, °)

**Table:**

*D*—H···*A*	*D*—H	H···*A*	*D*···*A*	*D*—H···*A*
O2—H2···O1	0.84	1.88	2.6058 (17)	144
O3—H3···O1	0.84	1.91	2.6267 (17)	142
O3—H3···O4^i^	0.84	2.50	2.9306 (15)	113
C15—H15···O1^ii^	0.95	2.57	3.3297 (18)	137

Symmetry codes: (i) −*x*+2, *y*−1/2, −*z*+1/2; (ii) −*x*+2, *y*+1/2, −*z*+1/2.
